# aPKC: the Kinase that Phosphorylates Cell Polarity

**DOI:** 10.12688/f1000research.14427.1

**Published:** 2018-06-25

**Authors:** Yang Hong

**Affiliations:** 1 Department of Cell Biology, University of Pittsburgh School of Medicine, S325 BST, 3500 Terrace Street, Pittsburgh, PA 15261, USA

**Keywords:** aPKC, Par-6, Par-3, apical-basal polarity, anterior-posterior polarity, epithelial cells, Drosophila, C. elegans, one-cell embryo, polybasic domain

## Abstract

Establishing and maintaining cell polarity are dynamic processes that necessitate complicated but highly regulated protein interactions. Phosphorylation is a powerful mechanism for cells to control the function and subcellular localization of a target protein, and multiple kinases have played critical roles in cell polarity. Among them, atypical protein kinase C (aPKC) is likely the most studied kinase in cell polarity and has the largest number of downstream substrates characterized so far. More than half of the polarity proteins that are essential for regulating cell polarity have been identified as aPKC substrates. This review covers mainly studies of aPKC in regulating anterior-posterior polarity in the worm one-cell embryo and apical-basal polarity in epithelial cells and asymmetrically dividing cells (for example, *Drosophila* neuroblasts). We will go through aPKC target proteins in cell polarity and discuss various mechanisms by which aPKC phosphorylation controls their subcellular localizations and biological functions. We will also review the recent progress in determining the detailed molecular mechanisms in spatial and temporal control of aPKC subcellular localization and kinase activity during cell polarization.

## Introduction

Atypical protein kinase C (aPKC), classic PKC (cPKC), and novel PKC (nPKC) are the three major PKC subfamilies ^1^. Unlike cPKC and nPKC, which are activated by calcium or diacylglycerol or both, aPKC is activated and regulated only by protein-protein interactions ^1^. Twenty years ago, the Ohno group, who were at the time searching for aPKC-interacting proteins, identified a mammalian protein they termed ASIP (aPKC-specific interacting protein), which turned out to be the homologue of *Caenorhabditis elegans* polarity protein Par-3 ^2^. Following this lead, they showed that worm aPKC (PKC-3) colocalizes with Par-3 at the anterior cortex of one-cell embryos and that RNA interference (RNAi) knock-down of aPKC gave *par-3*-like anterior-posterior (A-P) polarity defects ^3^. Furthermore, PKC-3 cortical localization is lost in *par-3* and *par-6* mutants and becomes symmetrical in *par-2* and *par-5* polarity mutants ^3^. These pioneering studies in mammalian cell culture and *C. elegans* for the first time established aPKC as a potential key polarity protein. Interestingly, *pkc-3* was not among the six *partition defective* ( *par*) mutants (that is, *par-1* to *par-6*) discovered by Kemphues’ group ^4^ in the seminal genetic screen on genes required for A-P polarity in worm one-cell embryos. Kemphues’ group, however, soon cloned worm *par-6* and found that Par-6 colocalizes with Par-3 and aPKC. In fact, all three proteins are dependent on each other for asymmetric cortical localization in one-cell embryos, suggesting that Par-3 (ASIP), aPKC, and Par-6 form a complex ^4^.

Par-6 was quickly recognized as an essential protein partner of aPKC, as its physical interaction with aPKC was confirmed simultaneously by several groups ^5– 7^. It is noteworthy that two of these studies independently discovered Par-6 on the basis of its physical interaction with Cdc42 through yeast two-hybrid screens ^6, 7^ and Cdc42 also turned out to be an important regulator of aPKC. Par-6 and aPKC bind each other through interactions between their N-terminal PB1 domains ^8^, and so far experimental evidence has been highly consistent that Par-6 and aPKC robustly associate and colocalize with each other *in vivo* (reviewed in 9) ^10^. In most cases, aPKC and Par-6 mutually require each other for their subcellular locations. Another aPKC partner protein p62 (also known as sequestosome 1 or SQSTM1) also binds to aPKC through PB1/PB1 interactions. However, the aPKC/p62 complex in general is not involved in regulating polarity but rather in the signaling pathways such as nuclear factor kappa B (NFκB) activation (reviewed in 11).

## Phosphorylation targets of aPKC in cell polarity

The role of aPKC, as a kinase, in regulating cell polarity centers primarily on its phosphorylation of various targets. It is fair to say that the list of aPKC substrates is long and distinguished and keeps growing. In this review, we can only briefly cover a short list of polarity or polarity-related proteins, including Lgl ^12– 15^, Numb ^16, 17^, Miranda (Mir) ^18^, Par-1 ^19– 22^, Par-2 ^23^, Pins ^24^, Baz/Par-3 ^25– 27^, Dlg ^28^, Par-6 ^29^, Crb ^30^, Yurt ^31^, Rock1 ^32^, and GSK3β ^33, 34^.

A recurring theme of aPKC phosphorylation-dependent regulation is that phosphorylation by aPKC often inhibits target proteins from localizing to plasma membrane (PM) or cell cortex allowing apically or anteriorly localized aPKC to exclude these target proteins from opposite PM/cortical domains during the process of establishing and maintaining polarity. Phosphorylation-dependent regulation of membrane/cortical localization of target proteins by aPKC may act through several mechanisms. First, phosphorylation by aPKC can directly inhibit a target protein from physically binding to PM. It has long been shown that phosphorylation by aPKC excludes Lgl, Numb, and Mir from the apical PM/cortex to maintain Lgl within the basolateral membrane in epithelial cells and Numb and Mir at the basal membrane in asymmetrically dividing neuroblasts. Mechanisms underlying this phosphorylation-dependent inhibition of PM/cortical localization of Lgl, Numb, and Mir had long been puzzling, and only recently has it become clear that Lgl, Numb, and Mir are all direct PM-binding proteins containing so-called polybasic (also known as “basic-hydrophobic”) domains which are highly positively charged because of the abundance of Arg and Lys residues ^14, 15^. Since the inner surface of PM is the most negatively charged membrane surface inside the cell because of its unique enrichment of polyphosphoinositides PI4P and PI(4,5)P _2_ (PIP _2_) ^35^, positively charged polybasic proteins can specifically target to PM through electrostatic interactions ^36– 39^. Moreover, critical aPKC phosphorylation sites on Lgl, Numb, and Mir all reside in their polybasic domains, enabling aPKC phosphorylation to neutralize the positive charges to directly prevent Lgl, Numb, and Mir from binding to PM ^14, 15^. Such charge-based and phosphorylation-dependent regulation actually is very similar to the well-characterized MARCKS protein, in which PM-binding polybasic effector domain (ED) is also inhibited by PKC phosphorylation ^40^. However, not all identified aPKC phosphorylation sites regulating PM localization of Numb and Mir are in polybasic domains; thus, mechanisms other than charge neutralization may also act to prevent polybasic domains from binding to PM. For instance, aPKC phosphorylation could induce conformation changes or protein interactions that hinder the polybasic domain from binding to PM. In addition, a recent study showed that although aPKC phosphorylation of the polybasic domain clears Mir from PM at interphase in asymmetrically dividing neuroblasts, at metaphase phosphorylation of the polybasic domain may actually enhance the actomyosin-dependent anchoring of Mir to basal PM ^41^.

Besides Lgl, Numb, and Mir, *C. elegans* Par-2 may also bind to PM through aPKC-regulated electrostatic interaction with phospholipids. In one-cell embryos, aPKC phosphorylates Par-2 to exclude it from the anterior cortex ^23^. Interestingly, Par-2 contains an Arg-rich cluster that is required for both *in vitro* binding to PIP _2_ and PI(3,4,5)P _3_ (PIP _3_) and *in vivo* association with PM/cortex ^42^. Moreover, phosphorylation by aPKC inhibits the binding of Par-2 to phospholipids *in vitro*, although the inhibition is unlikely due to direct charge neutralization, as the identified aPKC phosphorylation site is outside the Arg cluster. Given that Par-2 PM localization does not require actomyosin cortex ^43^, it is plausible that Par-2 directly binds to PM through electrostatic interaction that can be inhibited by aPKC phosphorylation.

aPKC phosphorylation could also inhibit target protein from binding to PM or cortex by inducing binding of scaffolding proteins to target proteins. In *Drosophila* and mammalian epithelial cells, Par-1 phosphorylation by apical aPKC inhibits the PM localization of Par-1 and excludes Par-1 from apical membrane ^20, 22^. In *Drosophila* oocyte and worm one-cell embryos, aPKC phosphorylation excludes Par-1 from the anterior cortex ^21, 23^. Such membrane exclusion likely involves phosphorylation-dependent binding of adaptor protein 14-3-3 (Par-5) ^20, 44^ that may potentially mask the PM-binding domain KA1 in Par-1 ^45^. Similarly, in MDCK cells, apically localized aPKC phosphorylates Pins to induce 14-3-3 binding that sequesters Pins from apical PM and therefore maintains the basolateral localization of Pins that is critical for orienting spindles horizontally during cell divisions ^24^.

In contrast to phosphorylation of Lgl, Numb, and Mir, phosphorylation of Baz/Par-3 and Dlg by aPKC controls their subcellular localization by modulating their interactions with other polarity proteins. Mammalian Par-3 contains a conserved C-terminal domain that can be bound and phosphorylated (at S827, S980 in Baz) by aPKC ^5, 46^. Biochemically, Baz is unique in the sense that it can act as both a substrate and an inhibitor of aPKC ^5^, but the molecular mechanisms switching the function of Baz between an inhibitor and a substrate of aPKC remain unclear. Recent studies showed that the CR3 domain carrying the aPKC phosphorylation site of Baz contains two flanking “arms” that bind to the kinase domain of aPKC and inhibit its kinase activity ^47^. Mutations in these arms turn Baz from an inhibitor into a good substrate of aPKC. In 2010, three groups reported that, in *Drosophila*, aPKC phosphorylation on S980 in Baz plays an epithelia-specific role to confine Baz localization at adherens junction (AJ) ^25– 27^. Phosphorylation of S980 inhibits the interaction of Baz with apical polarity protein complexes aPKC/Par-6 or Sdt/Crb or both, allowing aPKC to clear Baz from apical PM and concentrate it to apical AJ. Supporting the idea that binding between Baz and aPKC helps to retain Baz at apical PM, non-phosphorylatable Baz-S980A shows expanded localization on apical PM while Baz-S980A carrying additional mutations in flanking arms of the CR3 domain that no longer binds aPKC remains at AJ. However, phosphomimetic Baz-S980E fully rescues the maternal and zygotic *baz* null mutant, suggesting that spatial and temporal regulations of aPKC phosphorylation on Baz are not essential. In addition, although expression of Baz-S980A at moderate levels in *baz*-deficient follicular cells caused apical constriction phenotypes, this phenotype could be neomorphic given recent studies showing that Baz is in fact dispensable for apical-basal (A-B) polarity in follicular cells ^48^, and follicular cells expressing only kinase-dead aPKC show normal Baz localization ^49, 50^. Overall, more studies are needed to understand the role of aPKC phosphorylation of Baz/Par-3 in A-B polarization *in vivo*.

Dlg is the latest member of the polarity protein family that was identified as a substrate of aPKC ^28^. In this case, though, aPKC phosphorylation does not act to control Dlg PM/cortical localization but instead regulates Dlg function in controlling spindle orientation during asymmetric cell division. Dlg is a so-called MAGUK protein containing three PDZ domains, a SH3 domain, and a GUK domain. The SH3 and GUK domains can intramolecularly or intermolecularly bind to each other into a self-inhibited conformation ^51^. Phosphorylation of SH3 domain by aPKC disrupts the interaction between SH3 and GUK domains, turning Dlg into an open and activated conformation capable of binding downstream effectors such as GuKH to orient spindle in asymmetric cell division.

Mechanisms by which aPKC regulates other phosphorylation targets in cell polarity are less clear. aPKC phosphorylates ROCK1 (Rho-associated kinase 1) to inhibit its localization at apical cell junctions in MDCK cells, preventing ROCK1 from inducing apical constriction ^32^. Phosphorylation of mammalian Par-6 by aPKC has been reported to promote epithelial-mesenchymal transition in mammalian cells ^29^; however, the identified phosphorylation site is not conserved in fly Par-6. In migrating cells, aPKC phosphorylates GSK3β to inactivate it at the leading edge, allowing adenomatous polyposis coli (APC)-dependent microtubule reorganization ^33, 34^. Finally, the intracellular domain of apical polarity protein Crumbs (Crb) was once shown to be a substrate of aPKC in *Drosophila* ^30^, and aPKC phosphorylation of Crb was further proposed to be essential for a feedback loop in polarizing Crb and Lgl subcellular localizations ^52^. However, non-phosphorylatable Crb knock-in mutants are homozygous-viable and have no discernable defects in polarity and development ^53^; so even if aPKC does phosphorylate Crb *in vivo*, these phosphorylations are dispensable.

## Regulation of aPKC subcellular localization during cell polarization

Given the myriad downstream targets that aPKC phosphorylates and regulates, how aPKC is regulated during cell polarization is obviously critical. Regulation of aPKC involves at least two important mechanisms. First, aPKC itself needs to be properly localized during polarization so that it can properly control target protein localization and activity. Second, during this process, aPKC kinase activity also needs to be tightly controlled to ensure that aPKC phosphorylates target proteins not only at the right localization but also at the right time.

How aPKC achieves polarized subcellular localization seems to be heavily cell context dependent. In *Drosophila* embryonic epithelial cells and neuroblasts as well as in worm one-cell embryos, apical/anterior localization of aPKC requires Baz/Par-3 ^3, 54– 56^. Physical interactions between aPKC and Baz/Par-3 are considered essential for this process, and two recent studies on the polarization of worm one-cell embryos further revealed intricate details in Par-3-dependent recruitment of aPKC and Par-6 to anterior membrane. Dickinson *et al*. developed an extremely sensitive sc-SiMPull (single-cell single-molecule pull-down) assay capable of quantifying Par-3, aPKC, and Par-6 proteins in individual complexes directly pulled from one-cell embryos ^10^. Their results demonstrated that the previously known oligomerization of Par-3 is essential for recruiting aPKC and Par-6 into large protein clusters that contain multiple Par-3, aPKC, and Par-6 proteins. The large size of these protein clusters makes them more efficiently transported by the actomyosin-based cortical flow during the establishment phase of A-P polarity in one-cell embryos, facilitating the relocalization of Baz, aPKC, and Par-6 to anterior membrane. It has also been speculated that cortical forces may stretch Par-3 to induce conformation changes that promote its oligomerization, hence the formation of the Par-3/aPKC/Par-6 cluster ^57^. However, during the maintenance phase of A-P polarity, PLK-1 kinase phosphorylates Par-3 to inhibit its oligomerization and subsequently resolves the large clusters of Par-3 and aPKC/Par-6 ^10^. This transition from cluster to more diffused localization likely releases more aPKC/Par-6 complexes from Par-3 and promotes their interaction with Cdc42 ^58^. Whereas Par-3 inhibits aPKC kinase activity, Cdc42 activates aPKC kinase activity to exclude posterior polarity proteins such as Par-1 and Par-2 from the anterior cortex (see below).

It should be noted that, although mechanisms revealed by these studies are elegant and detailed, the role of Baz in regulating aPKC localization is not universal. For instance, in *Drosophila* follicular cells, aPKC localization to apical PM is Baz independent, and the loss of aPKC localization in *baz* mutant cells shown by previous studies is likely due to additional background mutations in the particular *baz* alleles used ^48^. Other proteins regulating aPKC/Par-6 subcellular localization but (owing to space limitations) not covered in this review include Crb/Sdt ^59^, Canoe ^60^, and Morg1 ^61^. Willin (FRMD6), a FERM domain protein, recruits aPKC to apical AJ in MDCK cells ^32^. In MDCK cells going through cyst formation in three-dimensional culture, lumen formation starts with apical enrichment of PIP _3_ phosphatase PTEN, which enriches PIP _2_ on apical PM by converting apical PIP _3_ to PIP _2_. PIP _2_ specifically attracts Annexin, which binds Cdc42, which in turn brings aPKC/Par-6 to apical membrane ^62^. Interestingly, aPKC localization in *Drosophila* follicular epithelial cells also requires PIP _2_, as in PI4P5K mutant *sktl* cells defective in PIP _2_ synthesis aPKC becomes mislocalized prior to the mislocalization of Baz ^63^. At present, it is unclear whether the mislocalization of aPKC is a direct or indirect consequence of loss of PIP _2_. Finally, potential delivery of aPKC via dynein/kinesin-based Rab11- or Rab35-mediated vesicle trafficking could also be critical for the subcellular localization of aPKC ^64, 65^.

## Control of aPKC kinase activity in establishing cell polarity

Besides proper subcellular localization, tight control of aPKC kinase activity is equally crucial for aPKC to regulate cell polarity. Like other PKC family members, aPKC protein alone is considered self-inhibited because of the binding between its pseudosubstrate region and kinase domain ^1, 66^. Although conceptually such self-inhibition provides a perfect mechanism for selectively activating aPKC in a spatial-temporal pattern during polarization, it has been shown that aPKC protein purified from Sf9 cells shows 10% kinase activity compared with the truncated kinase domain, which is considered 100% active ^66^. Thus, aPKC has quite a high basal kinase activity, which also needs to be properly inhibited. However, experimental evidence regarding the roles of Par-6 and Cdc42 in regulating aPKC kinase activity has yielded conflicting models. Based on *in vitro* kinase assays, the Ohno group suggested that Par-6 binds to aPKC and such binding both inhibits aPKC kinase activity and potentiates aPKC activation when inhibition is released upon subsequent binding of Cdc42 to Par-6 ^67^. Similar results were shown by studies using purified aPKC/Par-6 complex from mammalian cells ^68^. These results support the pivotal role of Cdc42 in controlling aPKC/Par-6 activity in establishing A-P polarity in worm one-cell embryos ^58^ and in recruiting aPKC/Par-6 during lumen formation ^62^. Recent studies also showed that Cdc42 activation promotes aPKC/Par-6-dependent apical expansion during cell junction formation ^69^.

Nonetheless, there is also evidence that binding of Par-6 may instead activate aPKC ^33, 70^ by inducing allosteric conformational changes in aPKC that displace the auto-inhibitory pseudosubstrate region from kinase domain, a process apparently independent of Cdc42 ^70^. There is additional evidence suggesting that Cdc42-dependent activation of aPKC may not hold true in all cell types. In *Drosophila* embryos expressing only mutant Par-6 that is defective in binding Cdc42, Lgl is still phosphorylated ^71^. Similarly, Baz remains phosphorylated in *Drosophila cdc42* mutant photoreceptors ^27^. In *C. elegans*, Cdc42 is not required for A-B polarization in embryonic epithelial cells, although it regulates the epithelial elongation process involving cell shape changes and junctional actin dynamics ^33, 72^. Tight junction formation in MDCK cells is sensitive to the disruption of aPKC/Par-6 interaction but not to the overexpression of dominant-negative Cdc42 ^73^.

One possible reason for these discrepancies is that assaying aPKC kinase activity was carried out mostly *in vitro* by using either purified proteins or immunoprecipitated aPKC complexes ^66– 68, 70, 74– 76^. As most aPKC substrates are membrane/cell cortex bound, the relevance of biochemically reconstituted kinase assay to the *in vivo* regulation of aPKC kinase activity probably needs to be considered carefully. Recent experiments using *in vitro* giant unilamellar vesicles (GUVs) suggested that membrane binding of either aPKC or target proteins such as Lgl ^77^ can be critical in regulating aPKC phosphorylation. For instance, Lgl bound to negatively charged GUVs appears to be more resistant to aPKC, suggesting that membrane binding makes polybasic domain in Lgl less accessible to aPKC for phosphorylation. Furthermore, association of aPKC with PM or cell cortex could also potentially modulate its kinase activity, as negatively charged membrane phospholipids such as PIP _3_ can directly stimulate aPKC kinase activity as suggested by *in vitro* assays ^78^. Also notable is that the range of aPKC kinase activity change is rather moderate in most *in vitro* kinase assays, oftentimes measured in less than twofold to fourfold of increase/decrease ^66– 68, 74– 76, 78^, in contrast to over 10- or 20-fold changes of activity seen in the activation of c/nPKC isoforms ^1^. Whether such moderate kinase activity changes (if accurate also *in vivo*) can explain the potent regulatory power of aPKC on target proteins remains to be fully investigated. It is noteworthy that, by using different purification and reconstitution methods, Graybill *et al*. showed that kinase activity of aPKC/Par-6 complex *in vitro* can be 10 times higher than aPKC alone ^70^, suggesting the possibility of drastic changes of aPKC kinase activity *in vivo*. Overall, it appears that mechanisms regulating aPKC kinase activity can be highly dependent on cell types and polarization processes, likely by involving different sets of regulators of aPKC.

In addition, *in vivo* aPKC studies have been carried out using mostly genetic and overexpression methods that in general are not capable of determining the precise role of aPKC kinase activity at specific stages of cell polarization. To this end, acute manipulation of aPKC activity *in vivo* would be highly useful but can be technically challenging. In *C. elegans*, such technical hurdles were recently overcome by Rodriguez *et al*. ^58^ by using temperature-sensitive aPKC ^ts^ mutant and drug inhibition in permeabilized embryos. These tools allowed experiments to acutely inhibit aPKC kinase activity at specific stages of one-cell embryo polarization, revealing phenotypes different from loss of aPKC protein assays by RNAi depletion ^58^. For instance, Par-6 remains on the membrane when aPKC kinase activity is acutely inhibited, in contrast to *aPKC-RNAi* embryos in which Par-6 is lost from membrane because aPKC and Par-6 are mutually dependent on each other for subcellular localizations. In addition, although aPKC is lost from membrane in *par-3*, *par-6*, or *cdc42-RNAi* embryos, the authors—by fusing aPKC to the C1B domain of PKCα—could acutely force membrane targeting of C1B-aPKC by adding phorbol ester (phorbol 12-myristate 13-acetate, or PMA) to mutant embryos. PMA-induced PM targeting of C1B-aPKC acutely removed Par-2 from membrane in *par-3-RNAi* embryos but not in *par-6* or *cdc42-RNAi* embryos. Experiments based on such elegant acute manipulation of aPKC kinase activity allowed the authors to directly demonstrate *in vivo* that Par-3 inhibits while Par-6 and Cdc42 are required for aPKC kinase activity in one-cell embryos. They proposed that aPKC/Par-6 complex may cycle through Par-3 cluster and Cdc42, a process that involves Par-3 cluster concentrating aPKC at the anterior PM and Cdc42 forming localized active Cdc42/aPKC/Par-6 complexes whose diffusive nature promotes aPKC phosphorylation on posterior polarity proteins. However, at present, direct experimental evidence supporting the cycling of aPKC between Par-3 and Cdc42 complexes remains to be established.

## Concluding remarks

In the past two decades, we have witnessed tremendous progress in revealing the important role of aPKC in cell polarity. Nonetheless, several key questions remain. First, although the list of aPKC targets keeps growing longer, understanding the details of discrete regulatory events induced by aPKC phosphorylation and integrating them into the dynamic cellular processes leading to cell polarization are still highly challenging. Second, conflicting results regarding the regulation of aPKC kinase activity need to be reconciled, hopefully by discovering new molecular mechanisms on spatial and temporal control of aPKC kinase activity during cell polarization. A major obstacle appears to be that, except for the kinase domain, no protein structure data are available for the whole aPKC protein, making it difficult to determine how aPKC undergoes necessary conformation changes upon binding to different regulatory proteins such as Par-6, Par-3, and Cdc42. Further complicating the issue is that aPKC also needs to be phosphorylated to become kinase-active ^1^, and few studies have queried this phosphorylation-based regulation of aPKC *in vivo* during cell polarization ^49^. In addition, there is evidence suggesting the kinase-independent function of aPKC in cell polarity ^49^, which has been much less explored. Finally, PM/cortical localization of aPKC is critical for its function ^79^, but more studies are needed to determine how aPKC gets localized to PM/cell cortex. For instance, aPKC PM/cortical localization is sensitive to hypoxia ^14^ and loss of phospholipids such as PIP _2_^63^, but mechanisms underlying such unexpected hypoxia/phospholipid sensitivity and its significance in aPKC regulation and function are currently unknown. Recent studies in *C. elegans* one-cell embryos using sc-SiMPull ^10^, acute pharmacological manipulation taking advantage of permeabilized embryos ^58^, and sophisticated live imaging and physical modeling ^57^ are exemplary in terms of delineating the molecular mechanisms controlling the aPKC function and kinase activity in A-P polarization. Adapting these approaches to other polarity model systems such as *Drosophila* epithelia may be challenging but will be highly useful. Further developing novel techniques such as optogenetic tools ^80– 82^ and establishing more sophisticated cell polarity model systems will certainly be a great help to advance our understanding of aPKC.
